# Distribution of *MEFV* gene mutations and R202Q polymorphism in the Serbian population and their influence on oxidative stress and clinical manifestations of inflammation

**DOI:** 10.1186/s12969-016-0097-1

**Published:** 2016-07-01

**Authors:** Jelena Milenković, Jelena Vojinović, Maruša Debeljak, Nataša Toplak, Dragana Lazarević, Tadej Avčin, Tatjana Jevtović-Stoimenov, Dušica Pavlović, Vladmila Bojanić, Maja Milojković, Gordana Kocić, Andrej Veljković

**Affiliations:** Institute of Pathophysiology, Faculty of Medicine, University of Niš, Bul. Zorana Đinđića 81, 18000 Niš, Serbia; Department of Pediatric Rheumatology, Clinical Center, Faculty of Medicine, University of Niš, Bul. Zorana Đinđića 81, 18000 Niš, Serbia; Unit for Special Laboratory Diagnostics, Bohoričeva 20, 1000 Ljubljana, Slovenia; Department of Allergology, Rheumatology and Clinical Immunology, University Children’s Hospital, University Medical Center; Faculty of Medicine, University of Ljubljana, Bohoričeva 20, 1000 Ljubljana, Slovenia; Institute of Biochemistry, Faculty of Medicine, University of Niš, Bul. Zorana Đinđića 81, 18000 Niš, Serbia

**Keywords:** Autoinflammation, Recurrent fevers, *MEFV* gene mutations, R202Q polymorphism, Oxidative stress

## Abstract

**Background:**

The Mediterranean fever (*MEFV*) gene codes for protein pyrin, one of the regulators of inflammasome activity in innate immune cells. Mutations in this gene are considered the primary cause of Familial Mediterranean fever, but are also found in other monogenic and multifactorial autoinflammatory diseases. The aim of the study was to determine if healthy carriers of *MEFV* gene mutations and R202Q polymorphism have clinical manifestations of inflammation and impaired oxidative stress parameters.

**Methods:**

One hundred DNA samples from healthy volunteers (13.3 ± 8.87 years of age (mean ± SD); range 2–35) were sequenced by ABI PRISM 310 automated sequencer (PE Applied Biosystems, Norwalk, USA). The Eurofever questionnaire was used to collect retrospectively medical history data. Oxidative stress was determined by measuring spectrophotometrically thiobarbituric acid reactive substances (TBARS) in plasma and erythrocytes, as well as advanced oxidation protein products in plasma. Superoxide dismutase (SOD) activity was determined by McCord and Fridovich method in plasma and erythrocytes, while the catalase erythrocyte activity was assessed using a catalase ELISA kit.

**Results:**

We found heterozygous carriers of K695R/N mutations in 5 %, E148Q/N mutations in 6 %, R202Q homozygous polymorphism in 10 % and heterozygous R202Q alterations in 45 % of healthy volunteers. The *MEFV* mutation carriers and R202Q polymorphism homozygotes reported significantly more often recurrent febrile episodes (*p* = 0.009), diffuse abdominal pain (*p* = 0.025), and malaise (*p* = 0.012) compared to non-carriers. Erythrocyte TBARS levels and plasma SOD activity were higher in persons with *MEFV* mutations and R202Q/R202Q (*p* = 0.03 and *p* = 0.049, respectively).

**Conclusions:**

Healthy individuals may bear E148Q and K695R *MEFV* gene mutations, as well as R202Q polymorphism in homozygous state. The determined gene alterations contribute to a subtle oxidative stress and may be associated with more frequent episodes of fever and unspecific inflammatory manifestations. An incomplete penetrance or variable expressivity of R202Q in populations of different ethnicity could influence the expression of autoinflammatory diseases phenotype.

## Background

The Mediterranean fever (*MEFV*) gene codes for the protein pyrin that is expressed in the cells of the innate immune system, especially granulocytes, monocytes, synovial fibroblasts and dendritic cells. Although the precise role of pyrin has not yet been clearly determined, it is one of the specific modulators of inflammasome, caspase-1 activation with interleukin-1β (IL-1β) production, activation of nuclear factor-kB and leukocyte apoptosis [1–4].

Clinical consequences of altered inflammasome regulation result from interleukin-1β effects and massive influx of activated leukocytes into tissues. Activated neutrophils and monocytes release proinflammatory cytokines, initiate acute-phase inflammatory response and undergo oxidative burst with an intense generation of reactive oxygen and nitrogen species via the NADPH oxidase complex and myeloperoxidase system [1–6].

The *MEFV* gene mutations are considered the primary cause of familial Mediterranean fever (FMF), the most common periodic fever syndrome and the prototype of monogenic autoinflammatory disease (AID). However, *MEFV* gene mutations are also frequently found in other AIDs, including Behcet’s disease, Henoch-Schönlein purpura, polyarteritis nodosa, ulcerative colitis, multiple sclerosis, etc. The knowladge about its role in the pathogenesis of these diseases is still obscure, but it is hypothesized that *MEFV* mutations could predispose to proinflammatory states [7–11].

Familial Mediterranean fever predominantly affects the populations of eastern Mediterranean origin, such as Turks, Jews, Arabs and Armenians. There is no official registry of FMF prevalence in Serbia, and its clinical diagnosis is very rare. The most frequent clinical manifestations of FMF are self-limited recurrent attacks of high fever (>38 °C) and sterile serositis (mostly peritonitis), synovitis and erysipeloid rash [1–3]. In the attack period, there are high levels of acute-phase reactants, leukocytosis and increased oxidative stress, evidenced by defective antioxidant defenses and enhanced oxidation products. The parameters usually return to normal in the remission period. However, in some patients, subclinical inflammation and oxidative stress are reported during the disease-free period as well [12–15].

In a large percentage of FMF patients, these five *MEFV* mutations are most frequently found: M680I, M694V, M694I and V726A in exon 10, and E148Q in exon 2. M694V homozygotes have a severe form of the disease, while mutations, such as E148Q and V726A, have a reduced penetrance and rarely cause any symptoms [2, 4, 16, 17].

The precise influence of R202Q alteration, in exon 2 of the *MEFV* gene, on inflammasome activity has been analysed additionaly. A high frequency of R202Q heterozygosity is detected in various populations and does not seem to cause any autoinflammatory effect in its carriers. Since this high carrier rate could not be explained by inbreeding or genetic drift, it is proposed to represent a selective advantage, a type of balancing selection, possibly toward increased innate immune response to a yet unidentified pathogen [3, [18–21]. In contrast, the frequency of R202Q homozygosity is very low and has been found to be associated with autoinflammatory manifestations in FMF patients that do not have any *MEFV* mutation or when R202Q is in compound heterozygous state with a mutation. This shows that despite its low frequency, the R202Q homozygosity has a high penetrance rate and suggests a potent dosage-dependent pro-inflammatory effect [15, 21–23].

The aim of the study was to determine if healthy carriers of *MEFV* gene mutations and R202Q polymorphism have clinical manifestations of inflammation and impaired oxidative stress parameters.

## Methods

The study was conducted in compliance with the Declaration of Helsinki and Good Clinical Practice (GCP) Guidelines. The relevant study documents were approved by an independent Ethics Committee and the informed consent forms were obtained from the participants.

This cross-sectional study was performed as an addition to the recently published study by Debeljak M. et al. [24] intended to evaluate the distribution of *MEFV* gene mutations in healthy populations of South-East Europe. One hundered healthy subjects (age > 2 years) or their parents have signed informed consent for blood samples to be collected for genetics and oxidative stress parameters analysis, as well as medical history data. The Eurofever project questionnaire [25, 26] was used to collect retrospectivelly the data about occurrence of inflammatory clinical manifestations during their life. The questionnaire is designed to recognize autoinflammatory syndromes and to collect information about clinical presentations in patients with a genetically confirmed diagnosis of AID or suspected cases on the basis of positivity of diagnostic criteria. A part of the questionnaire about clinical manifestations describes the characteristics of disease episodes for a recurrent disease course, with detailed analysis of fevers (frequency, duration, triggers, etc.), muco-cutaneous manifestations, musculoskeletal system, lymphoid organs and other organ systems, constitutional symptoms (fatigue, malaise, weight loss, insomnia, etc.), surgical procedures, family history, laboratory findings (routine and specific blood examinations), imaging and drug therapy. Recurrent fever was defined as 3 or more episodes of fever (≥38.0 °C) in a 6 months interval, that lasted for a few days without any identified cause after thorough examination, followed by a fever-free interval (with a sense of well-being) of at least 2 weeks. Clinical manifestations were compared between the persons with determined *MEFV* mutations and R202Q polymorphism and the control group that comprised all the persons without *MEFV* mutations.

Healthy volunteers can be considered as representative of Serbian population since questionnaire and blood samples were obtained when subjects were performing routine blood tests for health check up. Inclusion criteria were absence of acute and chronic illnesses, fever free period of at least 2 weeks prior to the blood samples collection, and a signed informed consent. Blood samples with EDTA were collected at the Department of Pediatric Rheumatology, Clinical Center in Nis, Serbia. 200 μl of the whole blood was used for DNA isolation by DNeasy Blood & Tissue Kit (QIAGEN Inc., Valencia, CA, USA). The DNA samples were tested for the presence of *MEFV* gene mutations within the multinational Eurofever Project (EAHC, Project No2007332) [24–26]. The *MEFV* gene exons 2 and 10 were amplified individualy by PCR. The exon 2 (360 bp) was amplified using the forward 5′-AAAACGGCACAGATGATTCCG-3′ and reverse 5′-AAGGGCCTGCACTCCTTC-3′ primers, and exon 10 (400 bp) by the following primers: forward 5′-AGCAGGAAGAGAGATGCAGTG-3′ and reverse 5′-TTGGAGACAAGACAGCATGG-3′. The exon 10 amplicons were primarily screened using Denaturing high performance liquid chromatography (dHPLC), for separation of those with normal nucleotide sequences (WAVE®Systems 4500 Series, Transgenomic, Inc., Omaha: NE, USA). Thereafter, sequencing was carried out using the Big Dye Terminator cycle sequencing kit and ABI PRISM 310 automated sequencer (PE Applied Biosystems, Norwalk, USA). The exon 2 amplicons were directly sequenced using the same method. The sequencing was performed at the Center for Medical Genetics, University Children’s Hospital Ljubljana, Slovenia. Obtained nucleotide sequences were compared with *MEFV* gene sequences at the [GenBank:NM_000243.2].

Complete blood count parameters were assessed using COULTER® AcT Diff Analyzer (Beckman Coulter Corporation, Hialeah, FL, USA). For C-reactive protein (CRP) and albumins concentration measurement we used fully automated Erba Mannheim XL600 analyzer (ERBA Diagnostics Mannheim Gmbh, Baden-Wurttemberg, Germany). Erythrocyte sedimentation rate (ESR) was assessed using the Westergren ESR method [27].

Lipid peroxidation was evaluated measuring thiobarbituric acid reactive substances (TBARS) in plasma and washed erythrocytes. TBARS in erythrocytes were assessed spectrophotometrically using the method by Jain et al. [28]. Trichloroacetic acid and tertiary butyl alcohol were added to erythrocytes in phosphate buffer (pH-7.4) forming the chromogen. The absorption was measured at 532 nm wavelength. TBARS concentration was expressed as nmol/g of hemoglobin. TBARS concentration in plasma was determined spectrophotometrically using the method by Andreeva et al. [29]. This method is based on the reaction of malondialdehyde (MDA) with thiobarbituric acid, at a high temperature and low pH. Measurement of MDA-TBA2 chromogen was than assessed at 532 nm wavelength.

Protein oxidation was measured spectrophotometrically by determination of advanced oxidation protein products (AOPP) in plasma, according to chloramine T solution, which in the presence of potassium iodide have absorbance at 340 nm [30].

Total superoxide dismutase (SOD) activity was measured in plasma and erythrocytes. The activities of both SODs were determined by measuring the inhibition of pyrogallol autoxidation [31]. This is a “negative” type of reaction, as we measure the decrease of nitroblue tetrazolium oxidation at the expense of pyrogallol autoxidation under alkaline conditions. One unit of SOD was defined as the amount of enzyme that causes 50 % inhibition of the pyrogallol autooxidation rate at 420 nm.

The catalase activity was determined in erythrocyte lysates using a catalase ELISA kit (Enzo Life Sciences, Inc., Farmingdale, NY 11735, USA), according to the manufacturer’s instructions.

The results were expressed as means ± standard deviations (SD) or medians ± interquartile range (IQR), as required. Statistical analysis was conducted using Chi-squared test for association (with Phi and Cramer’s V test for the strength of association (Phi)), with statistical significance at *p* < 0.05, and Fisher’s exact test when necessary. The Hardy-Weinberg principle was applied to test for R202Q polymorphism. Conditional logistic regression analysis for matched data was used to estimate an interaction between genotype and clinical manifestations (outcome). Statistical analysis of oxidative stress parameters was conducted using the Mann-Whitney *U*-test. For correlation between the parameters we used Spearman’s rank order correlation. Statistical analyses were performed using SPSS 17.0 (SPSS, Chicago, IL, USA) statistical program.

## Results

Out of 100 healthy volonteeers enrolled, 47 were males and 53 females, with average age of 13.3 ± 8.87 years (mean ± SD) (range 2–35). All study subjests were Caucasians of the Serbian ethnicity, without family history of FMF or other autoinflammatory disease and no consanguinity. No demographic differences were determined between persons with different *MEFV* genotype.

The DNA samples sequencing has found that 11 % (*n* = 11) of subjects have *MEFV* gene mutations. No homozygous mutation for *MEFV* gene was determined. We have only found heterozygous *MEFV* gene mutation K695R/N (c.2084A > G) in exon 10 in 5 % (*n* = 5) of subjects, and E148Q/N (c.442G > C) mutation in exon 2 in 6 % (*n* = 6) of subjects. These subjects comprised the group we named “MEFV mutation carriers”.

The R202Q (c.605G > A) homozygous polymorphism was found in 10 % (*n* = 10) of subjects, and we named this group “R202Q homozygotes”. Only one subject had heterozygous K695R/N mutation in combination with R202Q homozygous state why we used his data for analysis in two groups (“MEFV mutation carriers” and “R202Q homozygotes”). Data for the groups *MEFV* mutation carriers and R202Q homozygotes were pulled together in larger group for more precise statistical analysis, and we named this group “MEFV/R202Qhomo”.

Heterozygous R202Q polymorphism was fond in 45 % (*n* = 45) of subjects, and they together with subjects with no *MEFV* mutations nor R202Q polymorphism (N/N) made the group named “control group” (*n* = 80) (N/N plus R202Q/N) (average age of 15.3 ± 11.6 years). No demographic differences were determined between subjects in different groups.

The allele and genotype frequencies for R202Q polymorphism were in Hardy-Weinberg equilibrium. However, the expected frequency of R202Q recessive phenotype (aa) was quite low: 10.6 % (f(a) = 0.325).

**Fig. 1:**
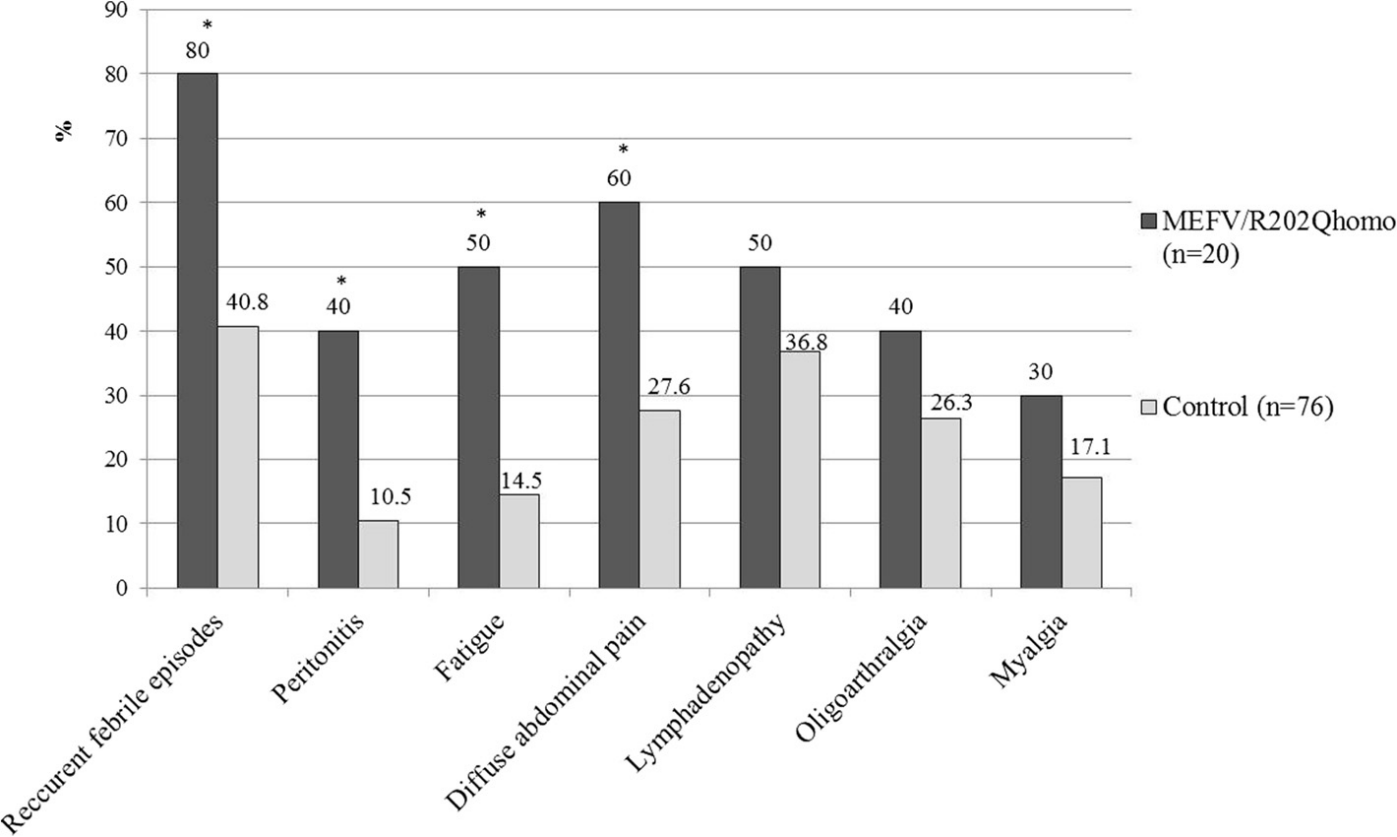
The significant difference of oxidative stress parameters in study subjects according to *MEFV* genotype group. **a** The TBARS concentrations in erythrocytes (median ± IQR) according to the *MEFV* genotype. *p = 0.03, compared to others. **b** Total SOD activity in plasma (mean ± SD) according to the *MEFV* genotype. *p = 0.001 and **p = 0.049, compared to others. MEFV/R202Qhomo - MEFV mutation carrier plus R202Q homozygotes group, Control group - N/N and R202Q/N

Erythrocyte TBARS levels were higher in MEFV/R202Qhomo group compared to the control group (*p* = 0.03, *U* = 84) (Fig. 1a). There was no difference in TBARS concentrations in plasma (*p* = 0.946, *U* = 161), nor AOPP levels (*p* = 0.322, *U* = 121), between the groups. There was positive corelation between AOPP and erythrocyte TBARS levels, but not statistically significant (*rs* = 0.643, *p* = 0.086). Plasma SOD levels were higher in MEFV/R202Qhomo group than in the control group (*p* = 0.049, *U* = 58.5) (Fig. 1b). Although the activities of erythrocyte SOD and catalase were lower in MEFV/R202Qhomo group, the difference was not statistically significant (for both groups *p* = 0.439). The R202Q homozygotes had significantly higher values of SOD plasma levels compared to the control group (*p* = 0.001) (Table 1). All the examined oxidative stress parameters did not show any important differences among subjects who were heterozygous for R202Q polymorphism and those without R202Q (N/N).

**Table 1:** Oxidative stress parameters according to the subjects genotype groups

Oxidative stress parameters	MEFV mutation carriers (*n* = 11)	R202Q homozygotes (*n* = 10)	MEFV/R202Qhomo (*n* = 20)	Control (*n* = 80)
TBARS in plasma (μmol/L)	2.36 ± 0.15	2.21 ± 0.12	2.327 ± 0.125	2.33 ± 0.46
TBARS in erythrocytes (nmol/gHb)	124.79 ± 46.41	132.09 ± 94.96	130.80 ± 68.86*	82.46 ± 34.16
AOPP (μmol/L)	40.15 ± 29.63	27.55 ± 17.59	32.63 ± 14.57	22.49 ± 20.24
SOD in plasma (U/ml)	3.61 ± 0.77	4.12 ± 0.46**	3.71 ± 0.48*	2.92 ± 0.96
SOD in erythrocytes (U/gHb)	10.37 ± 7.44	9.017 ± 2.09	9.02 ± 7.44	12.95 ± 7.54
Catalase in erythrocytes (U/ml/mgHb)	62.66 ± 44.04	75.71 ± 15.66	65.86 ± 23.98	80.13 ± 36.76

Values are shown as mean ± SD or median ± IQR

*MEFV/R202Qhomo* MEFV mutation carrier plus R202Q homozygotes group, *Control* N/N and R202Q/N, *TBARS* thiobarbituric acid reactive substances, *AOPP* advanced oxidation protein products, *SOD* superoxide dismutase

*p < 0.05; **p < 0.001

None of the subjects met clinical diagnostic criteria for FMF or other autoinflammatory diseases. In order to evaluate clinical manifestations in our subjects according to the *MEFV* genotype, we compared clinical manifestations frequency between defined groups (Table 2). Data was missing for 4 subjects in control group (N/N plus R202Q/N), for different manifestations and their data were excluded.

**Table 2:** The absolute and relative frequencies of most common clinical manifestations in study subjects according to *MEFV* genotype group

Clinical manifestations	MEFV mutation carriers (*n* = 11)	R202Q homozygotes (*n* = 10)	MEFV/R202Qhomo (*n* = 20)	Control (*n* = 76)
Reccurent febrile episodes	8 (72.7 %)*	8 (80 %)*	16 (80 %)**	31 (40.8 %)
Peritonitis	5 (45.5 %)**	3 (30 %)	8 (40 %)**	8 (10.5 %)
Fatigue/malaise	6 (54.5 %)**	4 (40 %)	10 (50 %)**	11 (14.5 %)
Diffuse abdominal pain	7 (63.6 %)*	6 (60 %)*	12 (60 %)**	21 (27.6 %)
Lymphadenopathy	3 (27.3 %)	7 (70 %)*	10 (50 %)	28 (36.8 %)
Oligoarthralgia	5 (45.5 %)	3 (30 %)	8 (40 %)	20 (26.3 %)
Myalgia	3 (27.3 %)	3 (30 %)	6 (30 %)	13 (17.1 %)

Values are shown as *n* (%)

*MEFV/R202Qhomo* MEFV mutation carrier plus R202Q homozygotes group, *Control* N/N and R202Q/N. Data for 4 subjects in control group were missing

*p < 0.05; **p ≤ 0.01 compared to control group

**Fig. 2:**
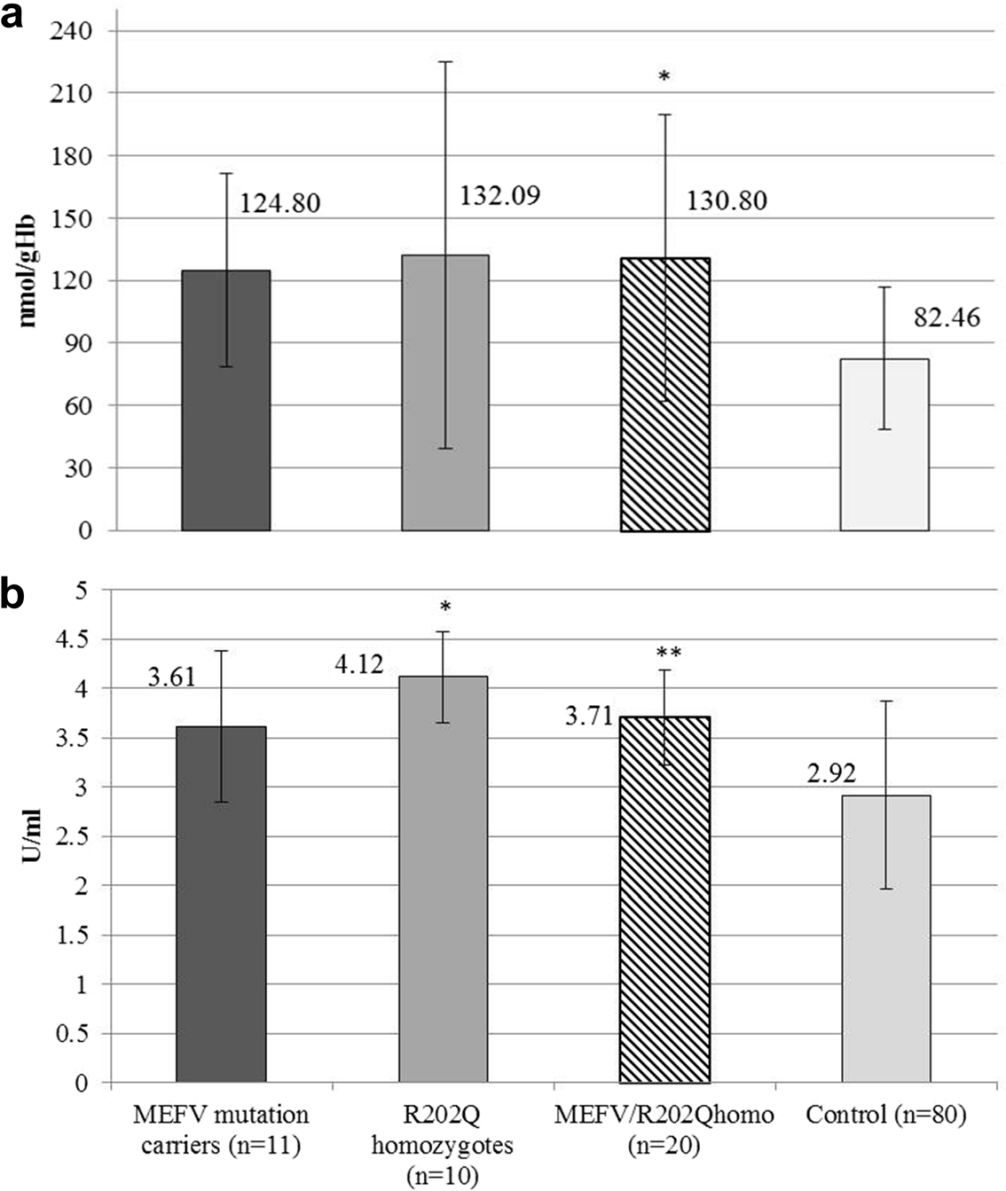
The clinical manifestations between mutation carriers with R202Q homozygotes and persons without *MEFV* gene changes. *p < 0.026. MEFV/R202Qhomo - MEFV mutation carrier plus R202Q homozygotes group, Control group - N/N and R202Q/N

The subjects in MEFV/R202Qhomo group reported significantly more often: recurrent febrile episodes of unknown cause (*p* = 0.002, *Phi* = 0.319), diffuse abdominal pain (*p* = 0.007, *Phi* = 0.277), peritonitis (*p* = 0.004, *Phi* = 0.321), and malaise/fatigue (*p* = 0.002, *Phi* = 0.349) compared to control group (Fig. 2). The results revealed a medium strong correlation between the occurrence of recurrent fever and determined heterozygous *MEFV* mutations and R202Q homozygosity. Average duration of fever episodes were 3.64 ± 1.26 days, and they reoccurred after 3.1 ± 1.2 months on the average.

The subjects in MEFV mutation carriers group experienced more often: recurrent febrile episodes of unknown cause (*p* = 0.048, *Phi* = 0.213), diffuse abdominal pain (*p* = 0.023, *Phi* = 0.256), peritonitis (*p* = 0.01, *Phi* = 0.326), and malaise/fatigue (*p* = 0.006, *Phi* = 0.336), compared to control group (*n* = 76). Subjects in R202Q homozygotes group, compared to control group, had significantly higher rates of recurrent fever (*p* = 0.022, *Phi* = 0.252), diffuse abdominal pain (*p* = 0.047, *Phi* = 0.224), and lymphadenopathy (*p* = 0.049, *Phi* = 0.216).

According to the conditional logistic regression analysis, no mutation nor R202Q alteration could significantly predict the development of most common clinical manifestations in our study group.

Complete blood count parameters and basic biochemical analyses (albumins, hemoglobin, CRP and ESR) were not different between the groups. There was a positive correlation between erythrocyte TBARS and leukocyte count (*rs* = 0.838, *p* = 0.009), as well as between plasma SOD levels and ESR (*rs* = 0.798, *p* = 0.031) in MEFV/R202Qhomo group.

## Discussion

The observed *MEFV* heterozygote mutation carrier rate of 11 % is relatively high for the Serbian healthy population. The clinical diagnosis of FMF, by applying Tel Hashomer criteria, is very rare in Serbia. It is possible that absence of FMF cases in Serbia is maybe due to the lack of the most commonly found mutations (M694V, V726A, M680I and M694I) and genotypes that are found in other populations in Mediterranean area. The mutations determined in our study were predominantly heterozygous E148Q and K695R. The E148Q mutation is frequently found in different populations and is usualy associated with milder clinical features with reduced penetrance. It is suggested to predispose to an inflammatory phenotype when in combination with other pro-inflammatory factors [32, 33]. In contrast to E148Q, K695R is considered a rare *MEFV* gene mutation. Generally, K695R has been found both as the sole mutation in FMF patients and in asymptomatic individuals. Because of its weak phenotypic presentation it has been supposed to predispose to subtle inflammatory changes [17, 20, 33–35]. We did not determine M694V mutation in our subjects and thus cannot say that there is linkage disequilibrium with the R202Q variant. The p.Ser52Asn of MVK and p.Gln703Lys of NLRP3 are not been analyzed and it would be interesting for further studies to investigate association of these changes [36].

It is interesting that in different persons the same *MEFV* mutation can be asymptomatic, it may present with inflammatory symptoms, or even be considered responsible for autoinflammatory disease [4, 16, 17]. Recognition of an inflammatory phenotype in single-mutation cases suggests that mutations lead to a gain-of-function with gene-dosage effect, rather than a simple loss-of-function recessive model, which has been considered their primary result [2, 18–20].

We observed a very high R202Q heterozygous carrier rate (45 %), as well as R202Q homozygosity in healthy individuals (10 %) compared to other studies, but without any clear influence on the phenotype. On the contrary, R202Q homozygosity was found in 9.2 % of Greek FMF patients [20] and in 43 % of Turkish FMF patients [22] in whom the phenotype corresponded to the characteristic autoinflammatory phenotype, with fever, serositis and monoarthritis occurring most frequently. Karakus et al. [37] showed that homozygous R202Q genotype was significantly higher in fibromyalgia patients than in healthy controls, and that morning fatique and irritable bowel syndrome had significant associations with this polymorphism.

In addition to the inflammatory symptoms, increased oxidative stress has been reported in AID patients during attacks, as well as in remission periods [14, 15]. It has been reported that neutrophils harboring *MEFV* gene mutations produce high levels of superoxide anion without any stimulation [6].

Since erythrocytes effectively scavenge reactive oxygen species, their membranes are simultaneously influenced by free radicals, intracellular antioxidative systems and plasma antioxidants. They are therefore regarded as a good indicator of lipid peroxidation [38]. The subjects in MEFV/R202Qhomo group in this study had significantly higher concentrations of erythrocytes TBARS, which might partly be the result of insufficient or depleted cellular antioxidant protection. The erythrocyte SOD and catalase activity were decreased in these persons but not statisticaly significant, and this should be reanalysed in a larger study group. It has been reported that FMF patients have significantly decreased activities of these enzymes in the attack period [13, 14]. Advanced oxidation protein products are novel oxidative stress and inflammation markers. Activated neutrophils are shown to participate directly in their formation [39]. There are no many studies evaluating the AOPP levels in FMF patients with *MEFV* mutations. Sahin et al. [40] determined lower AOPP levels in FMF patients with attack-free period than in controls. They studied AOPP levels according to the paraoxonase phenotype and found that FMF patients with a QQ phenotype exhibited lower AOPP levels than the controls, while there was not significant difference if the patients had QR + RR paraoxonase phenotype. However, comparably, free thiol levels and protein carbonyl groups are changed in the attack periods, decreased and increased respectively [13, 14]. Although not statistically significant, AOPP levels were increased in MEFV/R202Qhomo group subjects and positively correlated with TBARS erythrocyte levels. This all implies on a complex oxidative regulation depending on various factors.

The major SOD isoenzyme in plasma is CuZn-extracellular SOD (EC-SOD), mostly secreted by endothelial cells [41, 42]. Leukocytes produce high levels of superoxide anion after activation and EC-SOD is the main enzymatic scavenger in the extracellular matrix. Compared to other antioxidative enzymes, induction and regulation of EC-SOD is governed by cytokines and not its substrate O_2_^−^ or other oxidants [43, 44]. Elevated plasma SOD levels were reported to reflect a response to increased oxidative stress in cancer patients and were implicated as a sensitive marker of inflammatory processes [45, 46]. Moreover, increased plasma SOD activity was observed in severe forms of acute appendicitis, indicating increased neutrophil infiltration and inflammation [47]. Considering inflammasome dysfunction in leukocytes with mutated pyrin, the increased plasma SOD activity in our study was perhaps an adaptive response to enhanced leukocyte activation and IL-1β release, since the leukocyte count was not significantly changed.

Most prevalent clinical manifestations in a FMF attack are fever and abdominal pain due to peritonitis [2, 3]. These symptoms are seen not only in homozygotes for the most common *MEFV* mutations, but also in compound heterozygote carriers [20, 34]. Healthy volunteers in our study could not be diagnosed as FMF or any other AID, but the mutation carriers and R202Q homozygotes reported recurrent fever and diffuse abdominal pain significantly more often than non-carriers. This could indicate that persons with heterozygous mutations and R202Q/R202Q changes had an impaired innate immunity response. Additionally, this might imply an increased risk of developing inflammatory conditions during life. Unfortunately, due to the cross-sectional design of our study, this can be only speculated about, and prospective (life time) clinical follow-up studies are therefore warranted.

However, neither mutation nor R202Q/R202Q could predict an inflammatory outcome in our study. The persons affected had occasional health problems that could not be significantly associated with the presence of detected gene changes. Additionally, there were no signs of subclinical inflammation. Types and frequency of clinical manifestations in R202Q homozygotes group were not different in comparison to the E148Q and K695R heterozygotes, pointing to the similar effect of this *MEFV* gene change. An influence of gene-dosage effect could be proposed, as R202Q heterozygosity was quite frequent but was not associated with a particular health problem.

Although R202Q homozygosity was found in patients with typical FMF presentation in other studies [21, 22] the symptoms reported by our R202Q homozygotes were mild, atypical and often without accompanying fever. This is perhaps the consequence of an incomplete penetrance or variable expressivity of R202Q in the Serbian population caused by the influence of additional genetic or environmental factors characteristic for this region.

## Conclusion

Healthy individuals may bear E148Q or K695R mutations of the *MEFV* gene, as well as R202Q alteration in the homozygous state in Serbia. An incomplete penetrance or variable expressivity of R202Q in populations of different ethnicity could influence the expression of autoinflammatory disease phenotypes. These gene changes may influence innate immunity and oxidative stress regulation. *MEFV* gene changes do not necessarily induce an autoinflammatory disease in the Serbian population, but may be responsible for more frequent occurrence of fever episodes and unspecific inflammatory manifestations in carriers.

## Abbreviations

AID, autoinflammatory diseases; AOPP, advanced oxidation protein products; CRP, C-reactive protein; ESR, erythrocyte sedimentation rate; FMF, familial Mediterranean fever; GCP, good clinical practice; IL-1β, interleukin-1β; IQR, interquartile range; MEFV, Mediterranean fever; SD, standard deviation; SOD, superoxide dismutase; TBARS, thiobarbituric acid reactive substances
